# Hunting Guild Mediates the Effects of Fecundity and Mortality on Sexual Size Dimorphism in Spiders

**DOI:** 10.1002/ece3.73731

**Published:** 2026-06-30

**Authors:** Mona Hosseini, Balázs Vági, Hunor Takács‐Vágó, Tamás Szűts, Tamás Székely, Oscar G. Miranda

**Affiliations:** ^1^ HUN‐REN‐DE Reproductive Strategies Research Group, Department of Evolutionary Zoology and Human Biology University of Debrecen Debrecen Hungary; ^2^ Biodiversity, Climate Change and Water Management Competence Centre University of Debrecen Debrecen Hungary; ^3^ Department of Zoology University of Veterinary Medicine Budapest Budapest Hungary; ^4^ Department of Ethology Eötvös Loránd University Budapest Hungary; ^5^ Centre for Evolution, Department of Life Sciences University of Bath Bath UK

**Keywords:** fecundity selection, hunting guild, movement, sex‐biased mortality, sexual size dimorphism, spiders

## Abstract

Sexual size dimorphism (SSD) arises from sex‐specific selection on body size, primarily through sexual selection, mortality‐driven selection on males, and fecundity selection on females. A key ecological factor that may shape both processes is foraging behaviour, which determines movement, predation exposure and resource acquisition. Spiders provide a useful model system to test this idea because they exhibit extensive ecological diversity in prey‐capture strategies, and previous work has linked hunting strategies to reproductive traits, body size and SSD. However, these relationships remain poorly resolved and rarely tested within a comparative mechanistic framework. Here, using a phylogenetic comparative analysis of 264 species across eight hunting guilds, we tested whether hunting guild predicts variation in SSD and modulates the relationship between SSD, sex‐biased mortality and fecundity. We used sex differences in pitfall trap catches, taken from the literature, as a proxy for sex differences in mobility and mortality. SSD differed markedly among guilds, with the strongest female bias occurring in orb‐web weavers. The relationship between SSD and sex‐biased mobility/mortality was context‐dependent, emerging in sheet weavers but not in ground hunters. In contrast, we consistently predicted female and male body size, but not SSD, with effect sizes varying across guilds. Together, these results suggest that SSD does not arise from universal mechanisms, but from guild‐specific sexual asymmetries associated with mobility, predation exposure, and resource access, linking foraging strategy to the macroevolution of body size differences between the sexes.

## Graphical abstract

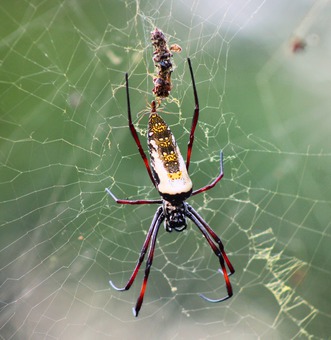

## 1 Introduction

Sexual size dimorphism (SSD), the difference in body size between males and females, evolves when selection on body size differs between the sexes (Darwin 1871; Price 1984; Slavenko et al. 2024). Although multiple selective forces may contribute to SSD evolution (e.g., Harvey and Dong 2023), two mechanisms are expected to play a central role across many animal groups: sex‐biased mortality and fecundity selection. First, differences in habitat use and mating‐related interactions can generate consistent differences in adult mortality, leading to sex‐specific selection on body size (Fairbairn et al. 2007; Lemaître et al. 2020). Second, female body size is positively associated with fecundity, with larger females often producing more offspring (Shine 1988; Honěk 1993), although the strength of this relationship varies across taxa (Slavenko et al. 2024).

A key ecological factor that may influence both sex‐biased mortality and fecundity selection is foraging behaviour. Given that the way individuals acquire food determines their energetic intake and their exposure to predators (Stephens and Krebs 1986; Lima and Dill 1990), if males and females differ in foraging behaviour, these differences may modulate sex‐biased mortality (e.g., Roy and Bhat 2018; Kienle et al. 2022) while also modifying the strength of fecundity selection on female body size (e.g., Gergely and Tökölyi 2023; Rocha and Gawryszewski 2024).

**FIGURE 1:**
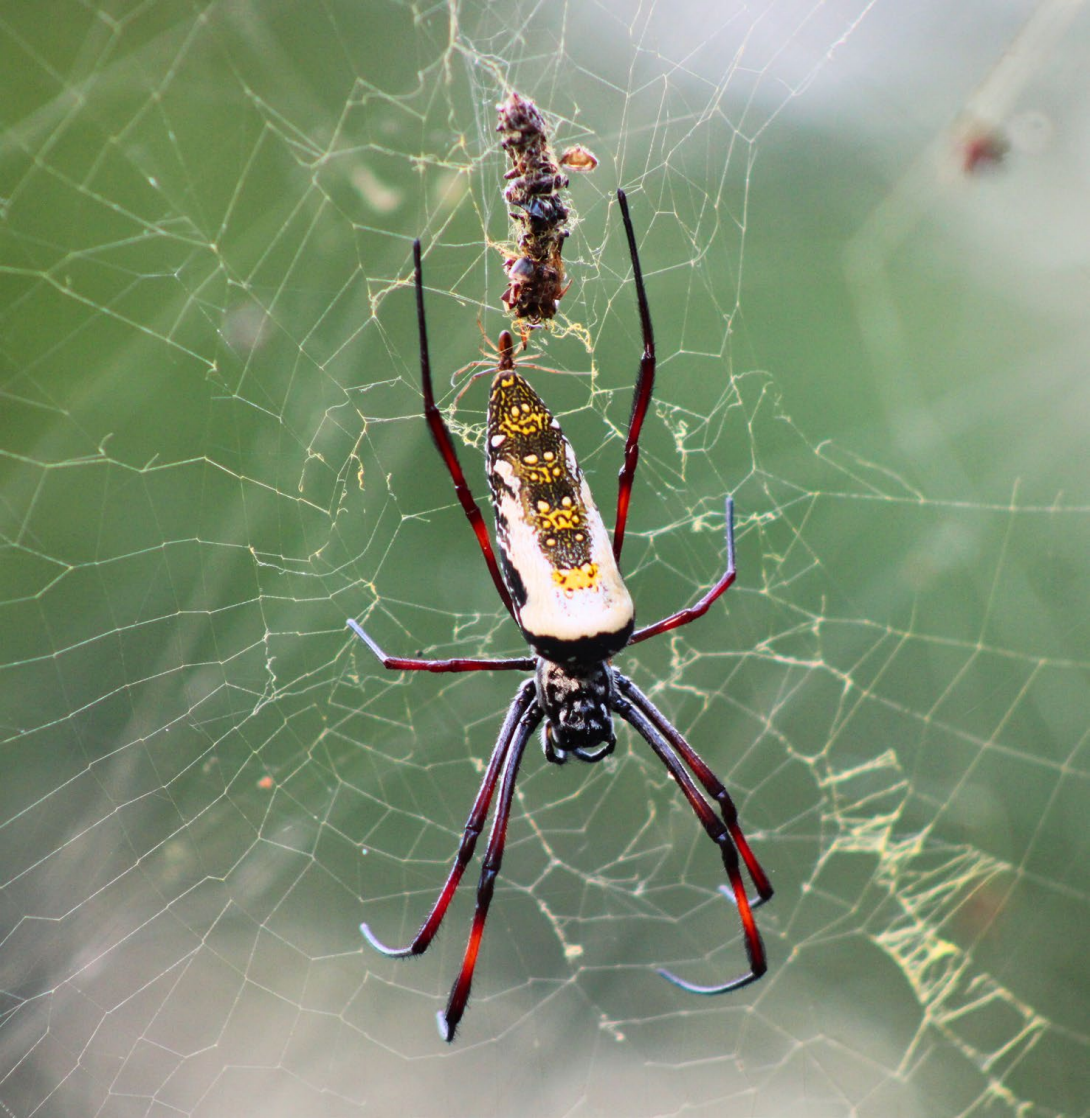
Female red‐legged golden orb‐weaver (*Trichonephila inaurata madagascarensis*) with a much smaller male visible near the tip of the female abdomen, southwest Madagascar. Orb‐web weavers are characterised by relatively sedentary females occupying aerial orb webs and highly mobile mate‐searching males. The pronounced sexual size dimorphism visible in this species illustrates the ecological asymmetries examined comparatively across spider hunting guilds in this study. Photograph by Oscar G. Miranda.

Spiders are a powerful model group for testing this idea because they exhibit diverse prey‐capture strategies and striking variation in sexual size dimorphism associated with hunting ecology (Figure 1; Cardoso et al. 2011). Some species build webs and remain sedentary, whereas others actively hunt or use sit‐and‐wait tactics, generating broad variation in predation risk and energy intake (Foelix 2025). Early work linked these foraging strategies—often termed ‘hunting strategies’—to reproductive traits and body size. For instance, Enders (1976) showed that web‐builders and non‐web spiders differ in egg production, suggesting that foraging strategies shape the strength of fecundity selection on body size.

Further, variation in prey‐capture strategies has long been proposed to explain SSD variation across spiders. Early comparative studies on SSD contrasted broad groups such as web‐builders, active hunters, and sit‐and‐wait species (Vollrath and Parker 1992; Head 1995), but remained largely descriptive. Later analyses incorporated phylogenetic methods, linking SSD to life‐history traits across hunters and sit‐and‐wait spiders, yet still relied on coarse ecological categories (Prenter et al. 1998). One of the last studies to explicitly examine SSD in relation to prey‐capture strategy was De Mas et al. (2009), who reported a negative relationship between SSD and sex‐biased mobility/mortality across active hunters and sit‐and‐wait spiders. Their estimates of sex‐biased mortality were derived from pitfall‐trap captures, which primarily reflect differential mobility between males and females, and indirectly, differences in predation exposure associated with movement. However, although their dataset included multiple prey‐capture strategies, hunting strategy itself was not tested as an ecological predictor mediating the relationship between SSD and sex‐biased mobility/mortality.

Beyond that, a key limitation of these studies is their composite hunting guild categorisation. Broad contrasts—web‐builders versus non‐web‐builders or sit‐and‐wait versus active hunters—mask substantial variation within categories. Among web‐builders, predation risk varies with web architecture (Craig 1987), with two‐dimensional web‐building spiders experiencing lower avian predation than three‐dimensional web‐builders (Gunnarsson and Wiklander 2015). Similarly, even within ambush‐hunting non‐web‐builders, differences in foraging strategies can alter energetic intake and potentially influence SSD evolution, as flower‐associated and visually deceptive crab spiders exhibit stronger female‐biased SSD than species relying solely on passive ambush predation (Rocha and Gawryszewski 2024).

A more functionally refined framework was proposed by Cardoso et al. (2011), who divided spiders into eight hunting guilds based on foraging strategy, prey range, vertical stratification and circadian activity. Because this framework groups multiple ecological dimensions simultaneously, hunting guild is likely to reflect differences in sex‐biased mobility, predation exposure and prey access among spider lineages (Cardoso et al. 2011). Different hunting guilds are expected to differ in the degree of sex‐biased mobility, which may influence sex differences in predation exposure and, consequently, mortality risk. At the same time, guilds also are expected to differ in prey access and energetic intake, factors that may affect the strength of fecundity selection.

Here, we use the hunting guild classification of Cardoso et al. (2011) in a phylogenetic comparative analysis to test whether hunting guild predicts variation in SSD and modulates the relationship between SSD, sex‐biased mobility/mortality and fecundity selection. We test three predictions. First, we expect guilds with strong male‐biased mobility to show more pronounced female‐biased SSD than guilds in which the sexes move more similarly, particularly where prey access allows females to attain larger size (e.g., orb‐web weavers compared to sheet‐ or space‐web weavers). Second, we expect the relationship between SSD and sex‐biased mobility to be steeper in guilds where males and females experience contrasting levels of predation exposure during foraging and mate searching (e.g., sheet‐web weavers, where males move extensively, compared to ground hunters, where both sexes are highly mobile). Third, we expect the relationship between SSD and fecundity to be steeper in guilds where females are relatively sedentary and experience consistent access to prey, allowing fecundity selection to favour larger body size (e.g., orb‐web weavers compared to active hunters or other guilds with less predictable foraging conditions).

## 2 Materials and Methods

### 2.1 Hunting Guild Classification

All species in the study were assigned to one of eight hunting guilds following the functional classification of Cardoso et al. (2011). Guild identity was determined at the family level and applied to all species within that family.

Guild identity was included as a categorical predictor in all analyses. Importantly, in this study hunting guild is treated as a functional proxy capturing consistent combinations of sex‐biased mobility, predation exposure and resource access. These underlying components are not directly quantified across all species but are inferred from empirically derived behavioural differences among guilds (Cardoso et al. 2011).

Guilds are briefly described below, based on hunting behaviour and activity patterns (Rose 2022):

- Sensing web weavers (e.g., Atypidae, Ctenizidae, Theraphosidae) are sedentary burrow‐dwellers that detect prey via silk tripwires; females show very low activity, whereas males undertake extensive mate‐searching.
- Sheet web weavers (e.g., Agelenidae, Linyphiidae) build horizontal sheets with funnel retreats, showing low overall activity and pronounced male‐biased movement.
- Space web weavers (e.g., Theridiidae, Pholcidae) construct irregular three‐dimensional cobwebs, remain near their webs, and exhibit moderate male roaming.
- Orb web weavers (e.g., Araneidae, Nephilidae, Tetragnathidae) build aerial orb webs that are periodically rebuilt; females are relatively sedentary, whereas males show strong mate‐searching mobility.
- Ambush hunters (e.g., Thomisidae, Sicariidae, Deinopidae) rely on concealment and short bursts of movement, with males ranging farther than females.
- Ground hunters (e.g., Lycosidae, Gnaphosidae, Corinnidae) actively pursue prey on the substrate and show high activity in both sexes and weak sex differences.
- Other hunters (e.g., Salticidae, Oxyopidae, Pisauridae) are visually guided stalkers with high activity in both sexes and little sex bias in movement.
- Specialists (e.g., Zodariidae, Mimetidae) target specific prey types and show variable but generally moderate activity and sex‐specific movement patterns.

### 2.2 Question 1: Does Hunting Guild Predict Sexual Size Dimorphism?

#### 2.2.1 SSD Data and Calculation

To test whether SSD varies across hunting guilds, we compiled sex‐specific adult carapace width and carapace length for 264 spider species from published sources. The full dataset, including trait measurements, hunting guild classifications, and associated literature references, is available in Dryad (Dataset DOI: 10.5061/dryad.95x69p918). Since body mass and abdomen length vary substantially with feeding status, hydration, and reproductive condition potentially obscuring fixed patterns of size dimorphism, we restricted analyses to carapace width and carapace length. Further, carapace dimensions are fixed after the final moult and provide a consistent estimate of structural body size (Foellmer and Moya‐Laraño 2007).

We quantified SSD using both carapace width and carapace length because structural body size in spiders cannot be fully captured by a single linear dimension. Morphometric studies routinely treat size and shape as distinct axes of variation, often combining carapace length and width to derive shape indices (e.g., width/length), while also analysing them separately (e.g., Wolff et al. 2022). Using both dimensions therefore reduces the risk that SSD estimates are driven by trait‐specific bias and allows consistent comparison across morphologically diverse taxa. We acknowledge, however, that linear measurements do not capture three‐dimensional body volume and may underestimate some aspects of size variation (Huber 2021).

To avoid ambiguity, we use ‘sexual size dimorphism (SSD)’ solely when referring to the general biological concept. For the quantitative indices derived from our dataset, we use carapace‐width dimorphism (CWD) and carapace‐length dimorphism (CLD), defined as log_10_(male/female). This provides a scale‐independent measure of proportional size difference between the sexes, where positive values indicate male‐biased and negative values indicate female‐biased dimorphism (Fairbairn et al. 2007).

#### 2.2.2 Phylogeny

We used the phylogenetic tree of Macías‐Hernández et al. (2020), pruned to match the species in our dataset. Phylogenetic signal in SSD was quantified using Blomberg's *K* (Blomberg et al. 2003) and Pagel's *λ* (Pagel 1999), implemented in the R packages ape (Paradis et al. 2004) and phytools (Revell 2012). Significance of *K* was assessed using 999 randomizations, and *λ* was evaluated via likelihood‐ratio tests against *λ* = 0.

#### 2.2.3 Statistical Model

We fitted phylogenetic generalised linear mixed models using the R package MCMCglmm (Hadfield 2010), with SSD (CWD and CLD) as response variables and hunting guild as a predictor.

Models assumed a Gaussian error distribution, and species identity was included as a random effect with a phylogenetic covariance structure using the inverseA() function. Weakly informative inverse‐Wishart priors (*V* = 1, *ν* = 0.002) were used.

Each model ran for 200,000 iterations, with a 50,000 burn‐in and thinning interval of 100, resulting in 1500 effectively independent posterior samples. Convergence diagnostics indicated stable chains (effective sample sizes > 1300, lag‐10 autocorrelations < 0.06, Geweke *z*‐scores within ±1.5; Geweke 1992).

### 2.3 Question 2: Does Hunting Guild Moderate the Effect of Sex‐Biased Mobility/Mortality on SSD?

#### 2.3.1 SSD Data

Sexual Dimorphism Indices (SDI) were obtained from De Mas et al. (2009), following Lovich and Gibbons (1992). We used SDI values based on carapace width and length only. The SDI divides the size of the larger sex by that of the smaller, subtracts one, and assigns negative values when males are larger.

#### 2.3.2 Sex‐Biased Mobility/Mortality Index

Sex‐biased mobility/mortality was also obtained from De Mas et al. (2009), calculated as the ratio of individuals of the more frequently trapped sex to the less frequently trapped sex in pitfall traps, with negative values indicating male bias.

The mortality dataset included 40 species, comprising two guilds with sufficient representation (14 ground hunters and 21 sheet‐web weavers).

#### 2.3.3 Phylogenetic Comparative Analyses

We used the Macías‐Hernández et al. (2020) phylogeny pruned to 40 species. Three models were fitted: one testing the direct association between mortality and SDI, a second incorporating hunting guild as an additive predictor, and a third assessing whether the effect of mortality on SDI differs between guilds through a mortality–guild interaction. The interaction term tests whether the relationship between mortality and SSD varies across guilds. All models were implemented in MCMCglmm using the same priors and diagnostics described above.

### 2.4 Question 3: Does Fecundity Predict SSD, and Is This Effect Moderated by Hunting Guild?

#### 2.4.1 Fecundity and SSD Data

Fecundity was quantified as clutch size (eggs per egg sac) from published sources (DOI: 10.5061/dryad.95x69p918). SSD was calculated as described above.

#### 2.4.2 Phylogeny for Fecundity Analyses

Because the Macías‐Hernández et al. tree lacked some taxa, we constructed a new phylogeny using COI sequences retrieved from GenBank (Benson et al. 2012). When COI sequences were unavailable for a focal species, we substituted the sequence of a congeneric species, provided it was the only representative of that genus in the dataset; otherwise, species without available sequences were excluded to preserve phylogenetic continuity within genera.

Sequences were aligned in Molecular Evolutionary Genetics Analysis version X (MEGA X; Kumar et al. 2018) using the MUSCLE algorithm with default parameters, and poorly aligned regions were trimmed. The phylogenetic tree was inferred using the Maximum Likelihood method under the Tamura–Nei (TN93) substitution model in MEGA X. The resulting tree was midpoint‐rooted, with branch lengths expressed in substitutions per site (uncalibrated). The final topology was exported for use in subsequent comparative analyses.

#### 2.4.3 Statistical Analyses

We first modelled SSD as a function of fecundity, log10‐transforming clutch size prior to analysis. These models included76 species for CWD and 53 species for CLD. We then tested whether hunting guild contributed additional explanatory power by fitting additive and interaction models including log10(clutch size), hunting guild, and their interaction. To ensure robust guild‐level estimates, these analyses included only hunting guilds represented by nine or more species: ground hunters, other hunters, and space‐web weavers for CWD, and ground hunters and space‐web weavers for CLD. Because one species ( *Cyrtophora moluccensis* ) produces exceptionally large clutches (> 1200 eggs; Berry 1987) relative to the remaining dataset, we additionally repeated the analyses excluding this species as a sensitivity analysis to assess whether the observed relationships were robust to a highly influential datapoint.

To increase statistical power and examine the sex‐specific components underlying SSD, we conducted parallel analyses using log_10_‐transformed male and female body size as response variables. These analyses tested whether log_10_(clutch size), hunting guild and their interaction predicted sex‐specific carapace width and carapace length. Models relating female body size to clutch size included 79 species for carapace width and 56 species for carapace length, whereas models relating male body size to clutch size included 76 species for carapace width and 53 species for carapace length. Guild‐level models retained only hunting guilds with sufficient representation to estimate guild effects. We also repeated the female and male carapace‐width analyses excluding *Cyrtophora moluccensis* as a sensitivity analysis. All models used the same MCMCglmm framework described above.

### 2.5 Statistical Inference

For each model, we report the posterior mean of the estimated effect size, along with the 95% Highest Posterior Density Interval (HPDI); intervals that do not cross zero indicate strong evidence for a real effect. The pMCMC value represents the Bayesian analogue of a *p*‐value, quantifying the probability that the estimated effect differs from zero. Model reliability was evaluated using the effective sample size of posterior draws (Eff.Samp), with higher values indicating more stable estimates. Explanatory power was summarised using the marginal *R* ^2^, which measures the proportion of variance explained by fixed effects.

Model fit was assessed using the Deviance Information Criterion (DIC), where lower values indicate a better trade‐off between fit and complexity.

All analyses were conducted in R version 4.3.1 (R Core Team 2024).

## 3 Results

### 3.1 Sexual Size Dimorphism Is Generally Weak Across Spiders

**FIGURE 2:**
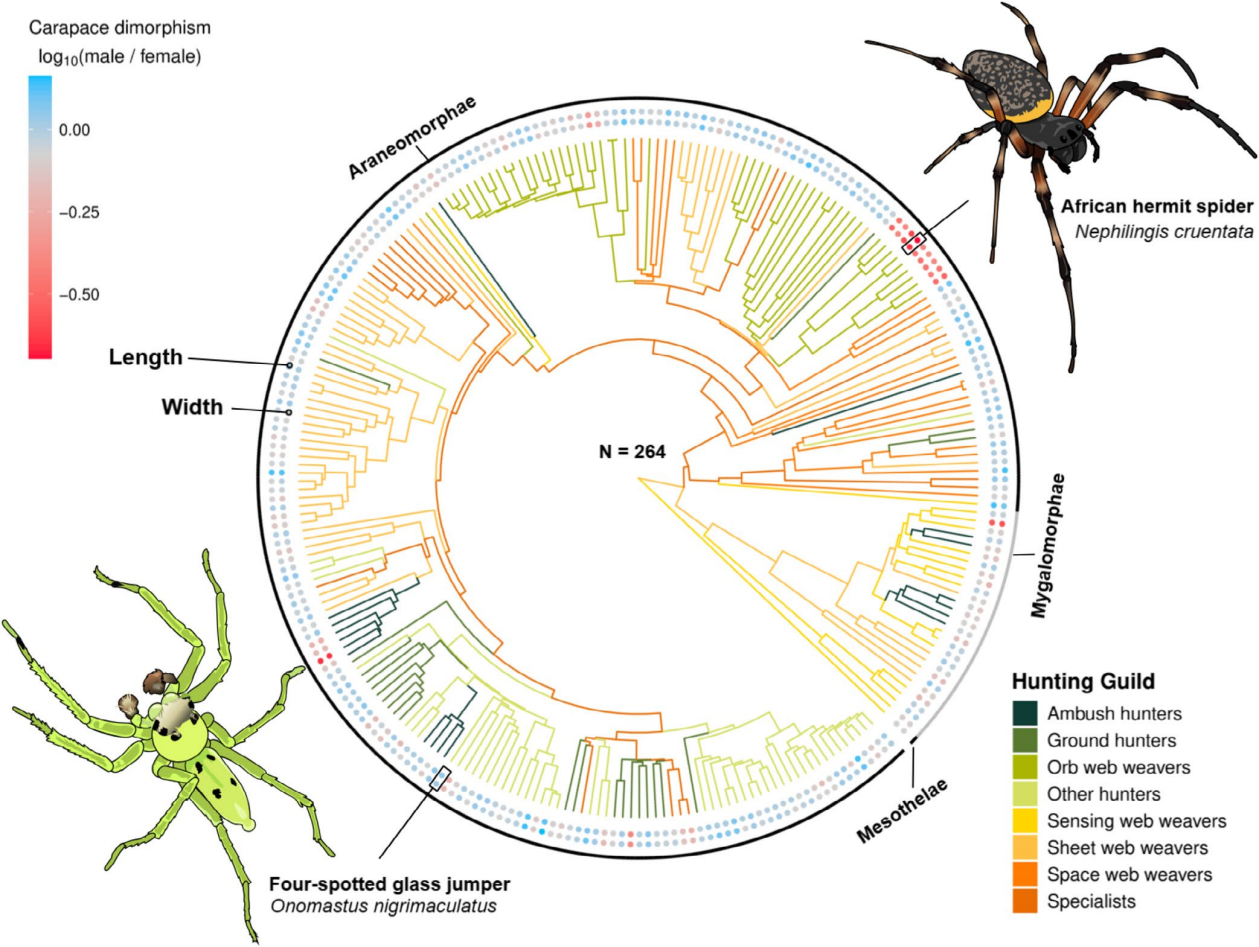
Phylogenetic distribution of carapace‐width dimorphism (CWD), carapace‐length dimorphism (CLD), and hunting guilds across 264 spider species. Branch colours denote hunting guilds following Cardoso et al. (2011). Outer dots represent species‐level CWD and inner dots represent CLD, both calculated as log_10_(male/female), where negative values indicate female‐biased dimorphism (red) and positive values indicate male‐biased dimorphism (blue). The three major lineages Mesothelae, Mygalomorphae and Araneomorphae are labelled. Two species are highlighted to illustrate the diversity of spider hunting guilds among our dataset: The African hermit spider ( *Nephilingis cruentata* ), an orb web weaver, and the four‐spotted glass jumper ( *Onomastus nigrimaculatus* ), a spider classified as other active hunter.

Across 264 spider species, carapace‐width dimorphism (CWD) was generally weak and slightly female‐biased (mean = −0.05, range = −0.65 to 0.13; Figure 2). CWD showed significant phylogenetic structure, although the strength of the signal depended on the metric used: Blomberg's *K* was relatively low (*K* = 0.315, *p* = 0.007), suggesting that closely related species were less similar than expected under Brownian motion, whereas Pagel's *λ* was high and significant (*λ* = 0.784, *p* < 0.001), indicating that shared ancestry still explains part of the covariance in SSD among species.

Carapace‐length dimorphism (CLD) showed a comparable distribution (mean = −0.05; range = −0.69 to 0.16) and a similar pattern of phylogenetic signal (*K* = 0.318, *p* = 0.007; *λ* = 0.770, *p* < 0.001).

Overall, SSD in spiders is generally weak but heterogeneous across species. The similar distributions and phylogenetic patterns obtained for CWD and CLD indicate that this conclusion is robust to the choice of body‐size metric.

### 3.2 SSD Varies Across Hunting Guilds Associated With Different Mobility and Foraging Contexts

**FIGURE 3:**
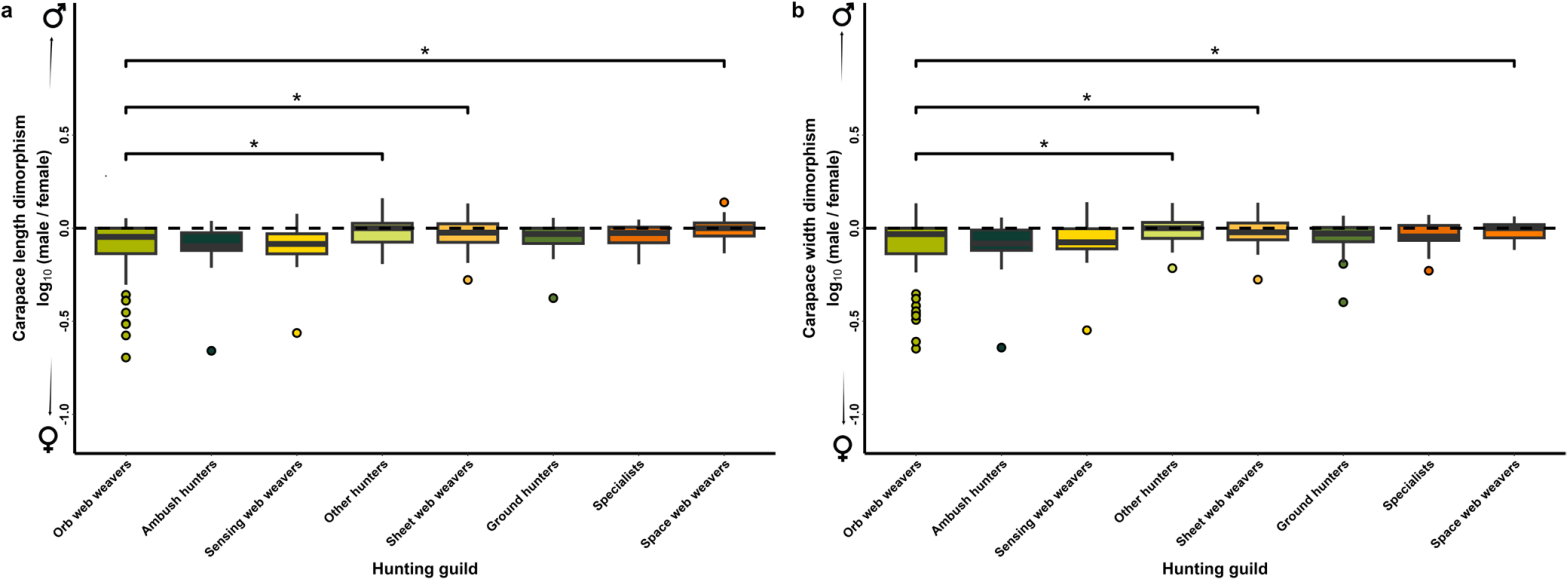
Carapace‐based dimorphism across ecological hunting guilds in 264 spider species. Panels show carapace‐length dimorphism (left, a) and carapace‐width dimorphism (right, b), both quantified as log_10_(male/female), so negative values indicate female‐biased dimorphism. The horizontal dashed line marks SSD = 0, corresponding to size monomorphism. Horizontal brackets indicate significant pairwise differences between hunting guilds based on MCMCglmm posterior mean contrasts; asterisks (*) indicate pMCMC < 0.05. (see Section 2). Sample sizes per guild are: 51 orb‐web weavers, 19 ambush hunters, 18 sensing web weavers, 52 other hunters, 62 sheet web weavers, 25 ground hunters, 17 specialists and 20 space web weavers. Hunting guilds follow Cardoso et al. (2011) classification.

CWD differed across hunting guilds (Figure 3), but differences were not evenly distributed.

Differences between orb‐web weavers and guilds in which both sexes exhibit high and similar activity levels were evident. Orb‐web weavers exhibited stronger female‐biased SSD than other hunters (CWD: –0.110 [–0.207 to –0.011], *pMCMC* = 0.032; Table S1). The same contrast was observed between orb‐web weavers and other hunters when using CLD (−0.103 [−0.204 to −0.014], *pMCMC* = 0.033; Table S2).

Differences were also evident within web‐building guilds. Sheet‐web and space‐web weavers, which construct more protected structures, showed weaker SSD than orb‐web weavers (CWD: posterior mean [95% CI] = 0.102 [0.017 to 0.186], *pMCMC* = 0.020; and posterior mean 0.092 [0.002 to 0.182], *pMCMC* = 0.048, respectively; Table S1), which build exposed aerial webs that allow them to access a wider range of resources. Analyses based on CLD recovered the same qualitative pattern (Table S2).

Notably, ambush hunters did not differ significantly from orb‐web weavers or from other web‐building guilds.

Overall, SSD is not uniformly associated with web‐building or with mobility alone but varies among guilds that combine different levels of sex‐biased mobility, exposure and resource access.

### 3.3 Hunting Guild Moderates the Effect of Sex‐Biased Mobility/Mortality on SSD

Across species, sex‐biased mobility/mortality showed a negative association with SSD, indicating that higher male‐biased mobility/mortality is associated with stronger female‐biased dimorphism. This relationship was supported in models on SDI‐CL (posterior mean [95% CI] = −0.211 [−0.382 to −0.027], *pMCMC* = 0.023) but was weaker and not statistically supported for SDI‐CW (Tables S3 and S4).

**FIGURE 4:**
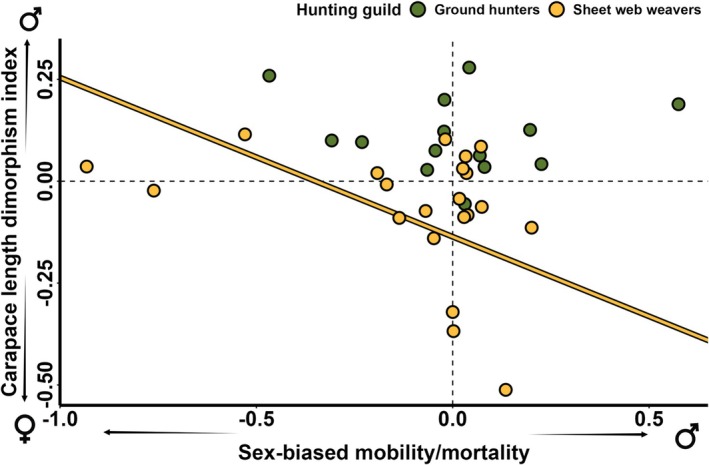
Relationship between sex‐biased mortality and the sexual dimorphism index (SDI) based on carapace length, using standardised values from De Mas et al. (2009). Points represent species means for two hunting guilds (yellow: Sheet‐web weavers, *n* = 21; green: Ground hunters, *n* = 14). SDI follows the index of Lovich and Gibbons (1992). The horizontal dashed line marks SDI = 0, indicating size monomorphism, and the vertical dashed line marks a mortality bias of zero, indicating equal male and female mortality. The solid line shows a phylogenetic generalised least‐squares regression fitted to sheet‐web weavers only and is included solely to illustrate the pattern detected in the MCMCglm models (see Section 3.3). These models revealed a marginally credible interaction between mortality bias and guild, driven by a strong negative mortality–SDI relationship in sheet‐web weavers and the absence of a detectable relationship in ground hunters.

Most importantly, when allowing the effect of mortality to vary across guilds, contrasting patterns emerged between guilds. In sheet‐web weavers, male‐biased mobility/mortality was strongly associated with increased female‐biased SSD (posterior mean = −0.358 [−0.572 to −0.162], *pMCMC* = 0.003; Figure 4; Table S4). In contrast, in ground hunters the relationship between mobility/mortality and SSD was effectively absent (posterior mean ≈0). This difference in slopes was reflected in the mobility/mortality × guild interaction term, which was supported in the CLD model (0.360 [0.006–0.716], *pMCMC* = 0.053; Table S4), but not in the CWD models.

### 3.4 Hunting Guild Shapes Fecundity‐related Body Size but Not Sexual Size Dimorphism

**FIGURE 5:**
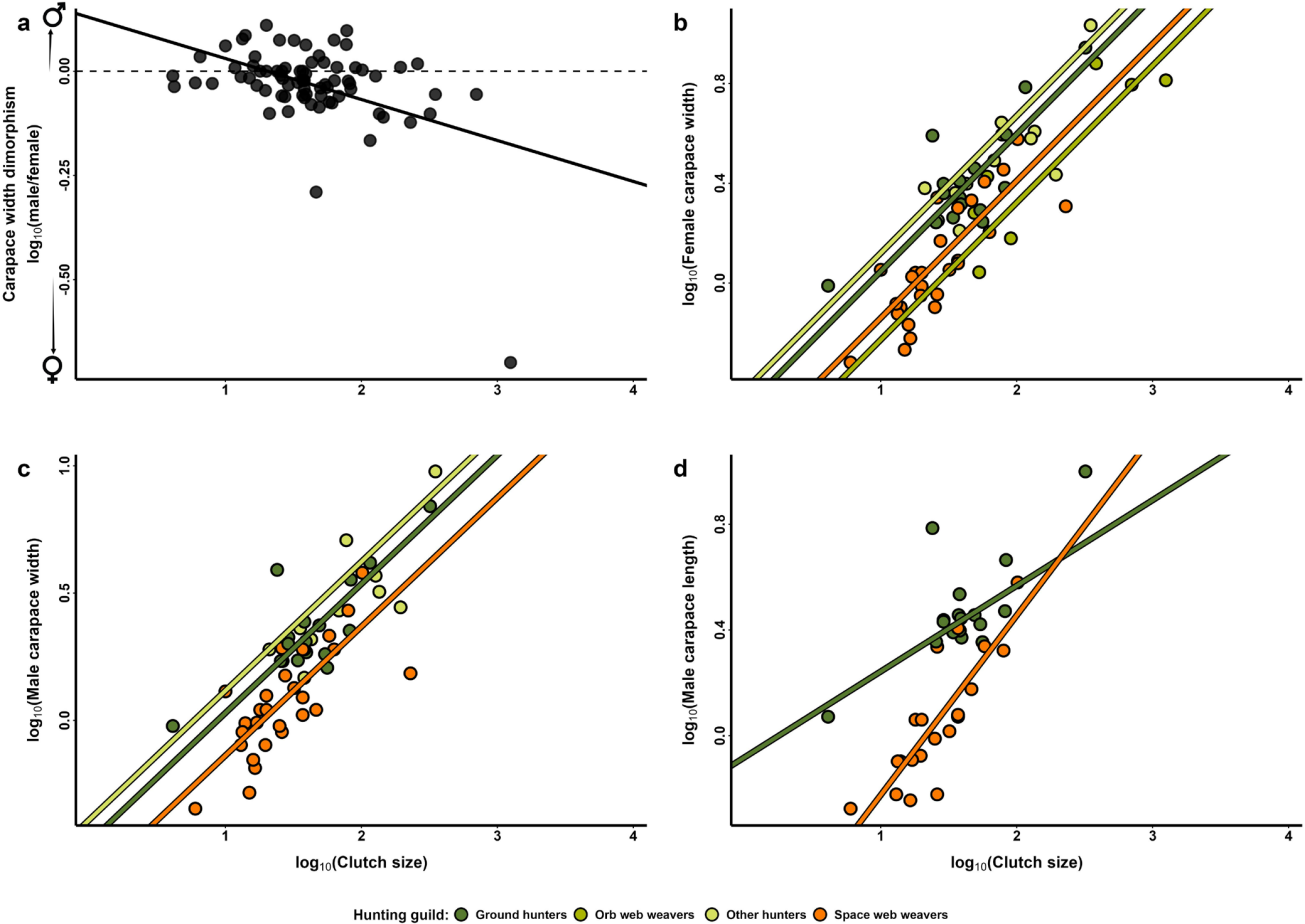
Associations between fecundity, sexual size dimorphism, and sex‐specific body size in spiders. (a) Relationship between carapace width dimorphism (CWD) and clutch size across 76 species. CWD is calculated as log_10_(male carapace width/female carapace width), where negative values indicate female‐biased dimorphism. The dashed horizontal line marks the point of size monomorphism. The solid regression line is a simple linear model shown for illustration only, to visualize the negative association detected in the MCMCglmm analysis (see Section 3.4). (b) Relationship between female carapace width, (c) male carapace width and (d) male carapace length, all log_10_‐transformed, and clutch size across spider species, coloured by hunting guild. Coloured regression lines are simple phylogenetic generalized least‐squares models presented for illustration of guild‐specific fecundity–size relationships. Panels (b) and (c) show that clutch size scales positively with both female and male carapace width, whereas panel (d) illustrates the guild‐specific relationship between clutch size and male carapace length. All regression lines are descriptive and are not the fitted MCMCglmm slopes. Symbol sizes are adjusted to highlight the hunting guilds involved in the main contrasts identified by the MCMCglmm models. See Section 3.4 for full model results.

Across 76 species, clutch size was negatively associated with carapace‐width dimorphism (CWD), indicating that species with larger clutches tended to show more female‐biased dimorphism (posterior mean = –0.103 [–0.156 to –0.054], *pMCMC* < 0.001; Table S5; Figure 5a). This relationship became weaker after excluding *C*. *moluccensis*, but remained negative (posterior mean = –0.040 [–0.074 to –0.001], *pMCMC* = 0.037; Table S6), indicating that clutch size still predicted CWD even without this extreme value. In contrast, clutch size was not clearly associated with carapace‐length dimorphism (CLD; posterior mean = –0.004 [–0.042 to 0.029], *pMCMC* = 0.823; Table S7). Hunting guild did not predict either CWD or CLD in any model, either directly or by modifying the relationship between clutch size and SSD, as both main effects and clutch size × guild interaction terms showed broad credible intervals overlapping zero (Tables [Link], [Link], [Link]).

To examine the sex‐specific components underlying SSD, we then tested whether clutch size predicted male and female body size. Clutch size was positively associated with female carapace width (posterior mean = 0.414 [0.321 to 0.520], *pMCMC* < 0.001; *n* = 79 species; Table S8), and this relationship remained robust after excluding *C*. *moluccensis* (posterior mean = 0.427 [0.306 to 0.544], *pMCMC* < 0.001; Table S9). Clutch size was also positively associated with female carapace length (posterior mean = 0.449 [0.309 to 0.606], *pMCMC* < 0.001; *n* = 56 species; Table S10). Similar patterns were detected in males. Clutch size was positively associated with male carapace width (posterior mean = 0.303 [0.175 to 0.414], *pMCMC* < 0.001; *n* = 76 species; Table S11), and this relationship remained after excluding *C*. *moluccensis* (posterior mean = 0.388 [0.275 to 0.506], *pMCMC* < 0.001; Table S12). Male carapace length was also positively associated with clutch size (posterior mean = 0.481 [0.331 to 0.645], *pMCMC* < 0.001; *n* = 53 species; Table S13). Hunting guild explained additional variation in sex‐specific body size. In additive models, space‐web weavers were generally smaller than ground hunters for female carapace width, female carapace length, male carapace width and male carapace length (Tables [Link], [Link], [Link], [Link], [Link], [Link]; Figure 5b,c). Other hunters did not differ clearly from ground hunters in most models. However, there was limited evidence that hunting guild modified the relationship between clutch size and body size. Interaction terms were not clearly supported for female or male carapace width (Tables [Link], [Link], [Link], [Link]; Figure 5b,c). However, for carapace length, the clutch size–body size slope tended to be steeper in space‐web weavers than in ground hunters, with weak support in females (clutch size × space‐web weavers: 0.303 [0.009 to 0.669], *pMCMC* = 0.065; Table S10) and clearer support in males (clutch size × space‐web weavers: 0.321 [0.027 to 0.641], *pMCMC* = 0.043; Table S13; Figure 5d).

## 4 Discussion

Across 264 species, SSD was generally weak, with strong female‐biased dimorphism concentrated in a subset of hunting guilds rather than distributed broadly across taxa. Three main patterns emerged. First, SSD differs among hunting guilds, with the strongest female bias occurring in orb‐web weavers. Second, the relationship between SSD and sex‐biased mortality is not general, but depends on hunting guild, emerging only in guilds where males and females differ strongly in movement, exposure and resource access. Third, fecundity did not consistently explain SSD directly, but hunting guild structured the fecundity–body size relationship, indicating that ecological mode influences whether reproductive output translates into sex‐specific size variation.

### 4.1 SSD Reflects Differences in Movement, Exposure and Resource Access

Orb‐web weavers showed stronger female‐biased dimorphism not only relative to highly mobile hunters, but also relative to other web‐building groups such as sheet‐web and space‐web weavers. These differences indicate that web‐building alone does not predict the SSD phenotype; instead, variation within web‐building guilds is critical (Craig 1987). This clarifies and refines the findings of Prenter et al. (1998), showing that the absence of differences among three foraging guilds (active hunters, ambush hunters and web‐builders) arises from within‐guild heterogeneity, rather than from a genuine lack of ecological structure in SSD.

In orb‐web weavers, females remain largely stationary on exposed webs, whereas males move extensively during mate searching. In contrast, in other hunters (e.g., Salticidae, Oxyopidae, Pisauridae) both sexes are highly mobile and likely experience similar exposure. Sheet‐web and space‐web weavers differ from orb‐web weavers in that their webs provide more protected structures, potentially reducing exposure despite similarly low female mobility. Further, the position of the orb‐web weavers might be in areas where they can capture more and larger prey.

### 4.2 Sex‐Biased Mobility/Mortality Explains SSD Only When Movement Differs Between Sexes

Across species, higher male‐biased mobility/mortality was associated with stronger female‐biased SSD, consistent with the original findings of De Mas et al. (2009), from which these data were derived.

However, our analyses show that this relationship depends on hunting guild. When allowing the effect of mobility/mortality to vary across guilds, the mobility/mortality–SSD relationship was strong in sheet‐web weavers, where males move substantially more than females, but absent in ground hunters, where both sexes exhibit similarly high levels of activity. This results are consistent with experimental evidence. Walker and Rypstra (2003), studying ground‐hunting spiders, found that differences in sex‐specific mobility did not translate into differences in mortality, matching our result that mortality does not predict SSD in this guild.

Finally, our results refine previous comparative work (Prenter et al. 1998) by suggesting that SSD is shaped not by body size interacting with foraging mode, but by sex‐biased mortality operating within specific hunting guilds.

### 4.3 Fecundity Predicts Body Size but Only Partially Scales to SSD

Fecundity was consistently associated with female body size across species, supporting previous work showing that larger females produce larger clutches (Head 1995; Prenter et al. 1999). However, this relationship was not restricted to females: Clutch size was also positively associated with male body size, indicating that fecundity is linked to broader body‐size scaling across species. Hunting guild further influenced this relationship, but only for carapace length, where the fecundity–size slope differed between ground hunters and space‐web weavers in both females and males. This is consistent with earlier observations that taxonomic groups, which are strongly associated with hunting guild (Enders 1976), differ in how fecundity translates into body size.

Although larger females consistently produced larger clutches, this relationship did not scale directly to dimorphism, challenging the widely held view that fecundity selection is the primary driver of SSD in spiders (Prenter et al. 1999). One possible explanation is that the extent to which fecundity selection can increase female size depends on ecological constraints associated with hunting guild. In more mobile female hunters, the demands of active foraging may limit extreme body enlargement despite the fecundity advantages of larger size, whereas more sedentary web‐building females may be able to attain larger body sizes while maintaining high reproductive output. However, because clutch size was also associated with male size, fecundity‐related body‐size scaling alone is insufficient to explain SSD. Under this interpretation, hunting guild may influence SSD not only through sex‐biased mobility/mortality, but also by mediating how fecundity relates to sex‐specific body size, particularly in carapace length.

### 4.4 Hunting Guild Structures SSD in Spiders: Implications and Limitations

Our results support the differential mortality hypothesis, which predicts that male dwarfism in any species with sit‐and‐wait female and wandering male life histories and a female‐biased operational sex ratio (Vollrath and Parker 1992; Kuntner and Coddington 2020). Nonetheless, differences in SSD among spiders can also arise through other mechanisms.

Particularly, differences in SSD among web‐building guilds may also be influenced by vertical structure, as proposed by the gravity hypothesis, which predicts that smaller males may be favoured because they can more efficiently reach females located on elevated webs (Corcobado et al. 2010). This could explain why orb‐web weavers exhibited stronger female‐biased SSD than sheet‐web weavers. However, this interpretation is not fully consistent with our results, as orb‐web weavers also differed from space‐web weavers, which similarly construct webs in elevated positions. Future studies should therefore quantify the actual distances males travel and the speed at which they reach females across web‐building guilds to clarify the role of vertical movement in shaping SSD.

Importantly, variation in male body size is unlikely to be explained solely by predation risk or locomotor constraints. Sexual selection processes not included in our analyses, particularly male–male competition and sexual cannibalism, are likely to contribute substantially to this variation (Wilder and Rypstra 2008a; Kwek et al. 2021). Smaller males may gain advantages by maturing earlier (protandry; e.g., Harvey et al. 2026), reaching females first, and reducing the risk of cannibalism. In contrast, larger males may benefit from increased competitive ability, allowing them to displace rivals or guard females, but at the cost of increased detectability and cannibalism risk. These opposing selective pressures suggest a trade‐off in male strategies, where the optimal body size depends on the balance between early access to mates, survival, and competitive success (Elgar and Fahey 1996).

The strength and direction of these pressures may also vary across hunting guilds. In guilds where prey capture is less reliable, consuming a male may provide a significant nutritional benefit that enhances female growth and fecundity, whereas in more effective hunting guilds this advantage may be reduced (Johnson 2001; Wilder and Rypstra 2008b). Consequently, male reproductive strategies are expected to coevolve with the ecological context defined by the female's hunting guild.

Taken together, our results suggest that hunting guild, by integrating sexual asymmetries associated with mobility, predator exposure and resource access, helps explain why the magnitude of SSD varies across spiders. More broadly, these findings are consistent with comparative studies in fishes, reptiles and birds showing that foraging mode can play a central role in shaping patterns of sexual size dimorphism across taxa (Butler et al. 2000; Krüger 2005; Pérez‐Camacho et al. 2018; Ronco et al. 2019).

## Author Contributions

**Mona Hosseini:** data curation (lead), formal analysis (lead), investigation (equal), visualization (equal), writing – original draft (lead). **Balázs Vági:** conceptualization (supporting), formal analysis (supporting), methodology (supporting), writing – review and editing (supporting). **Hunor Takács‐Vágó:** data curation (supporting), writing – review and editing (supporting). **Tamás Szűts:** investigation (equal), supervision (supporting), writing – review and editing (supporting). **Tamás Székely:** conceptualization (equal), funding acquisition (lead), methodology (lead), supervision (supporting), writing – review and editing (supporting). **Oscar G. Miranda:** conceptualization (equal), data curation (supporting), formal analysis (supporting), investigation (equal), methodology (supporting), supervision (lead), writing – review and editing (lead).

## Funding

This work was supported by HUN‐REN‐Debrecen University Reproductive Strategies Research Group (1102207) (T.Sk., B.V., O.G.M.), NKFIH Hungary (no. 150852; PD‐132819) (T.Sk., B.V.), HU‐RIZONT Hungary (no. 2024‐00109) (T.Sk.), Stipendium Hungaricum (2024_787527) (M.H.), Strategic research fund of the University of Veterinary Medicine 587 Budapest (no. SRF‐003) (T.Sz.), and University of Bath's University Research Studentship Award (E4G0BU9M57T0P0) (O.G.M.).

## Ethics Statement

This study did not involve any experiments on animals or humans. All data analysed were obtained from publicly available, open‐access sources that complied with the original authors' ethical standards. Therefore, no ethical approval or informed consent was required for this research.

## Conflicts of Interest

The authors declare no conflicts of interest.

## Supporting information


**Data S1:** Carapace_dataset.

**Data S2:** Carapace_dataset_tree.

**Data S3:** Carapace_Fecundity_data.

**Data S4:** Carapace_Fecundity_tree.

**Data S5:** Carapace_Mortality_data.

**Data S6:** Carapace_Mortality_tree.

**Data S7:** Hosseini_Ecology&SSDinSpiders_MainWorkspace.

**Table S1:** Pairwise contrasts of hunting guild effects on carapace width dimorphism (CWD) from MCMCglmm models. Shown are posterior means, 95% credible intervals and pMCMC values for all pairwise differences in CWD among the eight hunting guilds. Each block uses one guild as the reference level, with contrasts indicating the estimated difference in CWD (reference—contrast). Negative values indicate that the reference guild has more female‐biased CWD than the contrast guild. Estimates were derived from a phylogenetically controlled MCMCglmm model with > 800 effective samples for all parameters. Eff.Samp = effective sample size. A number of species per guild are as follows: Ambush hunters 19, Ground hunters 25, Orb web weavers 51, Other hunters 52, Sensing web weavers 18, Sheet web weavers 62, Space web weavers 20, Specialists 17.

**Table S2:** Pairwise contrasts of hunting guild effects on carapace length dimorphism (CLD) from MCMCglmm models. Shown are posterior means, 95% credible intervals and pMCMC values for all pairwise differences in CLD among the eight hunting guilds. Each block uses one guild as the reference level, with contrasts indicating the estimated difference in CLD (reference—contrast). Negative values indicate that the reference guild has more female‐biased CLD than the contrast guild. Estimates were derived from a phylogenetically controlled MCMCglmm model with > 1100 effective samples for all parameters. Eff.Samp = effective sample size. A number of species per guild are as follows: Ambush hunters 19, Ground hunters 25, Orb web weavers 51, Other hunters 52, Sensing web weavers 18, Sheet web weavers 62, Space web weavers 20, Specialists 17.

**Table S3:** Summary of MCMCglm model results for the sexual dimorphism index calculated from carapace width (SDI‐CW) against the sex‐biased mobility/mortality index and hunting guild. Three model formulations were tested: (1) SDI‐CW as a function of sex‐biased mortality (*N* = 40), (2) as a function of sex‐biased mortality and hunting guild (additive effects) and (3) including the interaction between sex‐biased mortality and hunting guild. We repeated Models 2 and 3 with (2a, 3a) Ground hunters, and with (2b, 3b) Sheet web weavers as the reference level. Only hunting guilds represented by nine or more species were included to ensure robust estimates. The table reports posterior means (post.mean), 95% credible intervals (low 95% CI–up 95% CI), pMCMC values, effective sample sizes (Eff.Samp) and residual variance, the marginal and conditional *R* ^2^ values, as well as the deviance information criteria (DIC). A number of species per hunting guild are as follows: Ground hunters = 14, Sheet web weavers = 21.

**Table S4:** Summary of MCMCglm model results for the sexual dimorphism index calculated from carapace length (SDI‐CL) against the sex‐biased mobility/mortality index and hunting guild. Three model formulations were tested: (1) SDI‐CL as a function of sex‐biased mortality (*N* = 40), (2) as a function of sex‐biased mortality and hunting guild (additive effects) and (3) including the interaction between sex‐biased mortality and hunting guild. We repeated Models 2 and 3 with (2a, 3a) Ground hunters, and with (2b, 3b) Sheet web weavers as the reference level. Only hunting guilds represented by nine or more species were included to ensure robust estimates. The table reports posterior means (post.mean), 95% credible intervals (low 95% CI–up 95% CI), pMCMC values, effective sample sizes (Eff.Samp) and residual variance, the marginal and conditional *R* ^2^ values, as well as the deviance information criteria (DIC). A number of species per hunting guild are as follows: Ground hunters = 14, Sheet web weavers = 21.

**Table S5:** Summary of MCMCglm model results for carapace width dimorphism (CWD) against hunting guild and clutch size. Carapace width dimorphism was calculated as log_10_(male carapace width/female carapace width). Clutch size was measured as number of eggs per egg sac, and it was log_10_‐transformed in all models. The table reports posterior means (post.mean), 95% credible intervals (low 95% CI–up 95% CI), pMCMC values, effective sample sizes (Eff.Samp), residual variance, the marginal and conditional *R* ^2^ values, as well as the deviance information criteria (DIC). Three model formulations were tested: (1) CWD as a function of clutch size (*n* = 76), (2) as a function of clutch size and hunting guild (additive effects) and (3) including the interaction between clutch size and hunting guild. We repeated Models 2 and 3 with Ground hunters (2a, 3a), with Other hunters (2b, 3b) and with Space web weavers (2c, 3c) as the reference level. Only hunting guilds represented by nine or more species were included to ensure robust estimates. A number of species per hunting guild are as follows: Ground hunters = 18; Other hunters = 11, Space web weavers = 27.

**Table S6:** Summary of MCMCglm model results for carapace width dimorphism (CWD) against hunting guild and clutch size. Carapace width dimorphism was calculated as log_10_(male carapace width/female carapace width). Clutch size was measured as number of eggs per egg sac, and it was log_10_‐transformed in all models. The table reports posterior means (post.mean), 95% credible intervals (low 95% CI–up 95% CI), pMCMC values, effective sample sizes (Eff.Samp), residual variance, the marginal and conditional *R* ^2^ values, as well as the deviance information criteria (DIC). Three model formulations were tested: (1) CWD as a function of clutch size (*n* = 75; without *Cyrtophora moluccensis*), (2) as a function of clutch size and hunting guild (additive effects) and (3) including the interaction between clutch size and hunting guild. We repeated Models 2 and 3 with Ground hunters (2a, 3a), with Other hunters (2b, 3b) and with Space web weavers (2c, 3c) as the reference level. Only hunting guilds represented by nine or more species were included to ensure robust estimates. A number of species per hunting guild are as follows: Ground hunters = 18; Other hunters = 11, Space web weavers = 27.

**Table S7:** Summary of MCMCglm model results for carapace length dimorphism (CLD) against hunting guild and clutch size. Carapace length dimorphism was calculated as log_10_(male carapace length/female carapace length). Clutch size was measured as number of eggs per egg sac, and it was log_10_‐transformed in all models. The table reports posterior means (post.mean), 95% credible intervals (low 95% CI–up 95% CI), pMCMC values, effective sample sizes (Eff.Samp), residual variance, the marginal and conditional *R* ^2^ values, as well as the deviance information criteria (DIC). Three model formulations were tested: (1) CLD as a function of clutch size (*n* = 53), (2) as a function of clutch size and hunting guild (additive effects) and (3) including the interaction between clutch size and hunting guild. We repeated Models 2 and 3 with Ground hunters (2a, 3a), and with Space web weavers (2b, 3b) as the reference level. Only hunting guilds represented by nine or more species were included to ensure robust estimates. A number of species per hunting guild are as follows: Ground hunters = 17; Space web weavers = 20.

**Table S8:** Summary of MCMCglm model results for female carapace width against hunting guild and clutch size. Female carapace width and clutch size (number of eggs per egg sac) were log_10_‐transformed in all models. The table reports posterior means (post.mean), 95% credible intervals (low 95% CI–up 95% CI), pMCMC values, effective sample sizes (Eff.Samp), phylogenetic and residual variance, the marginal and conditional *R* ^2^ values, as well as the deviance information criteria (DIC). Three model formulations were tested: (1) Female carapace width as a function of clutch size (*n* = 79), (2) as a function of clutch size and hunting guild (additive effects) and (3) including the interaction between clutch size and hunting guild. We repeated Models 2 and 3 with Ground hunters (2a, 3a), Orb web weavers (2b, 3b), Other hunters (2c, 3c) and with Space web weavers (2d, 3d) as the reference level. Only hunting guilds represented by nine or more species were included to ensure robust estimates. A number of species per hunting guild are as follows: Ground hunters = 18, Orb web weavers = 9, Other hunters = 11, Space web weavers = 27.

**Table S9:** Summary of MCMCglm model results for female carapace width against hunting guild and clutch size. Female carapace width and clutch size (number of eggs per egg sac) were log_10_‐transformed in all models. The table reports posterior means (post.mean), 95% credible intervals (low 95% CI–up 95% CI), pMCMC values, effective sample sizes (Eff.Samp), phylogenetic and residual variance, the marginal and conditional *R* ^2^ values, as well as the deviance information criteria (DIC). Three model formulations were tested: (1) Female carapace width as a function of clutch size (*n* = 78; without *Cyrtophora moluccensis*), (2) as a function of clutch size and hunting guild (additive effects) and (3) including the interaction between clutch size and hunting guild. We repeated Models 2 and 3 with Ground hunters (2a, 3a), Orb web weavers (2b, 3b), Other hunters (2c, 3c) and with Space web weavers (2d, 3d) as the reference level. Only hunting guilds represented by eight or more species were included to ensure robust estimates. A number of species per hunting guild are as follows: Ground hunters = 18, Orb web weavers = 8, Other hunters = 11, Space web weavers = 27.

**Table S10:** Summary of MCMCglm model results for female carapace length against hunting guild and clutch size. Female carapace length and clutch size (number of eggs per egg sac) were log_10_‐transformed in all models. The table reports posterior means (post.mean), 95% credible intervals (low 95% CI–up 95% CI), pMCMC values, effective sample sizes (Eff.Samp), phylogenetic and residual variance, the marginal and conditional *R* ^2^ values, as well as the deviance information criteria (DIC). Three model formulations were tested: (1) Female carapace length as a function of clutch size (*n* = 56), (2) as a function of clutch size and hunting guild (additive effects) and (3) including the interaction between clutch size and hunting guild. We repeated Models 2 and 3 with Ground hunters (2a, 3a), Other hunters (2b, 3b), and with Space web weavers (2c, 3c) as the reference level. Only hunting guilds represented by nine or more species were included to ensure robust estimates. A number of species per hunting guild are as follows: Ground hunters = 17, Other hunters = 8, Space web weavers = 20.

**Table S11:** Summary of MCMCglm model results for male carapace width against hunting guild and clutch size. Male carapace width and clutch size (number of eggs per egg sac) were log_10_‐transformed in all models. The table reports posterior means (post.mean), 95% credible intervals (Low 95% CI ‐ Up 95% CI), pMCMC values, effective sample sizes (Eff.Samp), phylogenetic and residual variance, the marginal and conditional *R* ^2^ values, as well as the deviance information criteria (DIC). Three model formulations were tested: (1) Male carapace width as a function of clutch size (*n* = 76), (2) as a function of clutch size and hunting guild (additive effects) and (3) including the interaction between clutch size and hunting guild. We repeated Models 2 and 3 with Ground hunters (2a, 3a), Other hunters (2b, 3b) and with Space web weavers (2c, 3c) as the reference level. Only hunting guilds represented by nine or more species were included to ensure robust estimates. A number of species per hunting guild are as follows: Ground hunters = 18, Other hunters = 11, Space web weavers = 27.

**Table S12:** Summary of MCMCglm model results for male carapace width against hunting guild and clutch size. Male carapace width and clutch size (number of eggs per egg sac) were log_10_‐transformed in all models. The table reports posterior means (post.mean), 95% credible intervals (low 95% CI–up 95% CI), pMCMC values, effective sample sizes (Eff.Samp), phylogenetic and residual variance, the marginal and conditional *R* ^2^ values, as well as the deviance information criteria (DIC). Three model formulations were tested: (1) Male carapace width as a function of clutch size (*n* = 75; without *Cyrtophora moluccensis*), (2) as a function of clutch size and hunting guild (additive effects) and (3) including the interaction between clutch size and hunting guild. We repeated Models 2 and 3 with Ground hunters (2a, 3a), Other hunters (2b, 3b), and with Space web weavers (2c, 3c) as the reference level. Only hunting guilds represented by nine or more species were included to ensure robust estimates. A number of species per hunting guild are as follows: Ground hunters = 18, Other hunters = 11, Space web weavers = 27.

**Table S13:** Summary of MCMCglm model results for male carapace length against hunting guild and clutch size. Male carapace length and clutch size (number of eggs per egg sac) were log_10_‐transformed in all models. The table reports posterior means (post.mean), 95% credible intervals (low 95% CI–up 95% CI), pMCMC values, effective sample sizes (Eff.Samp), phylogenetic and residual variance, the marginal and conditional *R* ^2^ values, as well as the deviance information criteria (DIC). Three model formulations were tested: (1) Male carapace length as a function of clutch size (*n* = 53), (2) as a function of clutch size and hunting guild (additive effects) and (3) including the interaction between clutch size and hunting guild. We repeated Models 2 and 3 with Ground hunters (2a, 3a), and with Space web weavers (2b, 3b) as the reference level. Only hunting guilds represented by nine or more species were included to ensure robust estimates. A number of species per hunting guild are as follows: Ground hunters = 17, Space web weavers = 20.

## Data Availability

The data and code are going to be available in the Dryad data repository upon acceptance. During review, the data are available for download here . https://doi.org/10.5061/dryad.95x69p918.

## References

[ece373731-bib-0001] Benson, D. A. , M. Cavanaugh , K. Clark , et al. 2012. “GenBank.” Nucleic Acids Research 41, no. D1: D36–D42. 10.1093/nar/gks1195. 23193287 PMC3531190

[ece373731-bib-0002] Berry, J. W. 1987. “Notes on the Life History and Behavior of the Communal Spider *Cyrtophora moluccensis* (Doleschall) (Araneae, Araneidae) in Yap, Caroline Islands.” Journal of Arachnology 15, no. 3: 309–319.

[ece373731-bib-0003] Blomberg, S. P. , T. Garland , and A. R. Ives . 2003. “Testing for Phylogenetic Signal in Comparative Data: Behavioral Traits Are More Labile.” Evolution 57, no. 4: 717–745. 10.1111/j.0014-3820.2003.tb00285.x. 12778543

[ece373731-bib-0004] Butler, M. A. , T. W. Schoener , and J. B. Losos . 2000. “The Relationship Between Sexual Size Dimorphism and Habitat Use in Greater Antillean *Anolis* Lizards.” Evolution 54, no. 1: 259–272. 10.1111/j.0014-3820.2000.tb00026.x. 10937202

[ece373731-bib-0005] Cardoso, P. , S. Pekár , R. Jocqué , and J. A. Coddington . 2011. “Global Patterns of Guild Composition and Functional Diversity of Spiders.” PLoS One 6, no. 6: e21710. 10.1371/journal.pone.0021710. 21738772 PMC3126856

[ece373731-bib-0006] Corcobado, G. , M. A. Rodríguez‐Gironés , E. de Mas , and J. Moya‐Laraño . 2010. “Introducing the Refined Gravity Hypothesis of Extreme Sexual Size Dimorphism.” BMC Evolutionary Biology 10, no. 1: 236. 10.1186/1471-2148-10-236. 20682029 PMC2924870

[ece373731-bib-0007] Craig, C. L. 1987. “The Significance of Spider Size to the Diversification of Spider‐Web Architectures and Spider Reproductive Modes.” American Naturalist 129, no. 1: 47–68. 10.1086/284622.

[ece373731-bib-0008] Darwin, C. 1871. The Descent of Man, and Selection in Relation to Sex. John Murray.

[ece373731-bib-0009] De Mas, E. , C. Ribera , and J. Moya‐Laraño . 2009. “Resurrecting the Differential Mortality Model of Sexual Size Dimorphism.” Journal of Evolutionary Biology 22, no. 8: 1739–1749. 10.1111/j.1420-9101.2009.01786.x. 19627415

[ece373731-bib-0010] Elgar, M. A. , and B. F. Fahey . 1996. “Sexual Cannibalism, Competition, and Size Dimorphism in the Orb‐Weaving Spider *Nephila plumipes* Latreille (Araneae: Araneoidea).” Behavioral Ecology 7, no. 2: 195–198. 10.1093/beheco/7.2.195.

[ece373731-bib-0011] Enders, F. 1976. “Clutch Size Related to Hunting Manner of Spider Species.” Annals of the Entomological Society of America 69, no. 6: 991–998. 10.1093/aesa/69.6.991.

[ece373731-bib-0012] Fairbairn, D. J. , W. U. Blanckenhorn , and T. Székely , eds. 2007. Sex, Size and Gender Roles: Evolutionary Studies of Sexual Size Dimorphism. 1st ed. Oxford University Press. 10.1093/acprof:oso/9780199208784.001.0001.

[ece373731-bib-0013] Foelix, R. F. 2025. Spider Biology. Springer.

[ece373731-bib-0014] Foellmer, M. W. , and J. Moya‐Laraño . 2007. “Sexual Size Dimorphism in Spiders: Patterns and Processes.” In Sex, Size and Gender Roles, edited by D. J. Fairbairn , W. U. Blanckenhorn , and T. Székely , 1st ed., 71–82. Oxford University Press. 10.1093/acprof:oso/9780199208784.003.0008.

[ece373731-bib-0015] Gergely, R. , and J. Tökölyi . 2023. “Resource Availability Modulates the Effect of Body Size on Reproductive Development.” Ecology and Evolution 13, no. 1: e9722. 10.1002/ece3.9722. 36620418 PMC9817193

[ece373731-bib-0016] Geweke, J. 1992. “Evaluating the Accuracy of Sampling‐Based Approaches to the Calculation of Posterior Moments.” In Bayesian Statistics 4, edited by J. M. Bernardo , J. O. Berger , A. P. Dawid , and A. F. M. Smith , 169–194. Oxford University Press. 10.1093/oso/9780198522669.003.0010.

[ece373731-bib-0017] Gunnarsson, B. , and K. Wiklander . 2015. “Foraging Mode of Spiders Affects Risk of Predation by Birds: Predation Risk in Spiders.” Biological Journal of the Linnean Society 115, no. 1: 58–68. 10.1111/bij.12489.

[ece373731-bib-0018] Hadfield, J. D. 2010. “MCMC Methods for Multi‐Response Generalized Linear Mixed Models: The MCMCglmm *R* Package.” Journal of Statistical Software 33, no. 2: 1–22. 10.18637/jss.v033.i02.20808728

[ece373731-bib-0019] Harvey, J. A. , and Y. Dong . 2023. “Climate Change, Extreme Temperatures and Sex‐Related Responses in Spiders.” Biology 12, no. 4: 615. 10.3390/biology12040615. 37106814 PMC10136024

[ece373731-bib-0020] Harvey, J. A. , F. Gerosa , R. Gols , and W. C. E. P. Verberk . 2026. “Growth, Development, and Survival in the Brown Widow Spider, *Latrodectus geometricus* , Under Different Feeding Regimes.” Journal of Arachnology 53, no. 3: 154–161. 10.1636/JoA-S-24-013.

[ece373731-bib-0021] Head, G. 1995. “Selection on Fecundity and Variation in the Degree of Sexual Size Dimorphism Among Spider Species (Class Araneae).” Evolution 49, no. 4: 776–781. 10.2307/2410330. 28565139

[ece373731-bib-0022] Honěk, A. 1993. “Intraspecific Variation in Body Size and Fecundity in Insects: A General Relationship.” Oikos 66, no. 3: 483. 10.2307/3544943.

[ece373731-bib-0023] Huber, B. A. 2021. “Beyond Size: Sexual Dimorphisms in Pholcid Spiders.” Arachnology 18, no. 7: 656–677. 10.13156/arac.2020.18.7.656.

[ece373731-bib-0024] Johnson, J. C. 2001. “Sexual Cannibalism in Fishing Spiders ( *Dolomedes triton* ): An Evaluation of Two Explanations for Female Aggression Towards Potential Mates.” Animal Behaviour 61, no. 5: 905–914. 10.1006/anbe.2000.1679.

[ece373731-bib-0025] Kienle, S. S. , A. S. Friedlaender , D. E. Crocker , R. S. Mehta , and D. P. Costa . 2022. “Trade‐Offs Between Foraging Reward and Mortality Risk Drive Sex‐Specific Foraging Strategies in Sexually Dimorphic Northern Elephant Seals.” Royal Society Open Science 9, no. 1: 210522. 10.1098/rsos.210522. 35116140 PMC8767210

[ece373731-bib-0026] Krüger, O. 2005. “The Evolution of Reversed Sexual Size Dimorphism in Hawks, Falcons and Owls: A Comparative Study.” Evolutionary Ecology 19, no. 5: 467–486. 10.1007/s10682-005-0293-9.

[ece373731-bib-0027] Kumar, S. , G. Stecher , M. Li , C. Knyaz , and K. Tamura . 2018. “MEGA X: Molecular Evolutionary Genetics Analysis Across Computing Platforms.” Molecular Biology and Evolution 35, no. 6: 1547–1549. 10.1093/molbev/msy096. 29722887 PMC5967553

[ece373731-bib-0028] Kuntner, M. , and J. A. Coddington . 2020. “Sexual Size Dimorphism: Evolution and Perils of Extreme Phenotypes in Spiders.” Annual Review of Entomology 65, no. 1: 57–80. 10.1146/annurev-ento-011019-025032. 31573828

[ece373731-bib-0029] Kwek, B. Z. W. , M. Tan , L. Yu , W. Zhou , C. C. Chang , and D. Li . 2021. “Aggressive Males Are More Attractive to Females and More Likely to Win Contests in Jumping Spiders.” Animal Behaviour 179: 51–63. 10.1016/j.anbehav.2021.06.030.

[ece373731-bib-0030] Lemaître, J. F. , V. Ronget , M. Tidière , et al. 2020. “Sex Differences in Adult Lifespan and Aging Rates of Mortality Across Wild Mammals.” Proceedings of the National Academy of Sciences of the United States of America 117, no. 15: 8546–8553. 10.1073/pnas.1911999117. 32205429 PMC7165438

[ece373731-bib-0031] Lima, S. L. , and L. M. Dill . 1990. “Behavioral Decisions Made Under the Risk of Predation: A Review and Prospectus.” Canadian Journal of Zoology 68, no. 4: 619–640. 10.1139/z90-092.

[ece373731-bib-0032] Lovich, J. E. , and J. W. Gibbons . 1992. “A Review of Techniques for Quantifying Sexual Size Dimorphism.” Growth, Development, and Aging 56: 269–281. 1487365

[ece373731-bib-0033] Macías‐Hernández, N. , M. Domènech , P. Cardoso , et al. 2020. “Building a Robust, Densely‐Sampled Spider Tree of Life for Ecosystem Research.” Diversity 12, no. 8: 288. 10.3390/d12080288.

[ece373731-bib-0034] Pagel, M. 1999. “Inferring the Historical Patterns of Biological Evolution.” Nature 401, no. 6756: 877–884. 10.1038/44766. 10553904

[ece373731-bib-0035] Paradis, E. , J. Claude , and K. Strimmer . 2004. “APE: Analyses of Phylogenetics and Evolution in R Language.” Bioinformatics 20, no. 2: 289–290. 10.1093/bioinformatics/btg412. 14734327

[ece373731-bib-0036] Pérez‐Camacho, L. , S. Martínez‐Hesterkamp , S. Rebollo , G. García‐Salgado , and I. Morales‐Castilla . 2018. “Structural Complexity of Hunting Habitat and Territoriality Increase the Reversed Sexual Size Dimorphism in Diurnal Raptors.” Journal of Avian Biology 49, no. 10: e01745. 10.1111/jav.01745.

[ece373731-bib-0037] Prenter, J. , R. W. Elwood , and W. I. Montgomery . 1998. “No Association Between Sexual Size Dimorphism and Life Histories in Spiders.” Proceedings of the Royal Society of London, Series B: Biological Sciences 265, no. 1390: 57–62. 10.1098/rspb.1998.0264.

[ece373731-bib-0038] Prenter, J. , R. W. Elwood , and W. I. Montgomery . 1999. “Sexual Size Dimorphism and Reproductive Investment by Female Spiders: A Comparative Analysis.” Evolution 53, no. 6: 1987–1994. 10.1111/j.1558-5646.1999.tb04580.x. 28565440

[ece373731-bib-0039] Price, T. D. 1984. “The Evolution of Sexual Size Dimorphism in Darwin's Finches.” American Naturalist 123, no. 4: 500–518. 10.1086/284219.

[ece373731-bib-0040] R Core Team . 2024. R: A Language and Environment for Statistical Computing. R Foundation for Statistical Computing. www.R‐project.org.

[ece373731-bib-0041] Revell, L. J. 2012. “Phytools: An R Package for Phylogenetic Comparative Biology (And Other Things).” Methods in Ecology and Evolution 3, no. 2: 217–223. 10.1111/j.2041-210X.2011.00169.x.

[ece373731-bib-0042] Rocha, P. N. , and F. M. Gawryszewski . 2024. “Foraging Strategy as a Route for Sexual Size Dimorphism Evolution.” Ecology and Evolution 14, no. 11: e70100. 10.1002/ece3.70100. 39512849 PMC11542996

[ece373731-bib-0043] Ronco, F. , M. Roesti , and W. Salzburger . 2019. “A Functional Trade‐Off Between Trophic Adaptation and Parental Care Predicts Sexual Dimorphism in Cichlid Fish.” Proceedings of the Royal Society B: Biological Sciences 286, no. 1909: 20191050. 10.1098/rspb.2019.1050. PMC6732390 31431167

[ece373731-bib-0044] Rose, S. 2022. Spiders of North America. Princeton University Press (Princeton Field Guides).

[ece373731-bib-0045] Roy, T. , and A. Bhat . 2018. “Population, Sex and Body Size: Determinants of Behavioural Variations and Behavioural Correlations Among Wild Zebrafish *Danio rerio* .” Royal Society Open Science 5, no. 1: 170978. 10.1098/rsos.170978. 29410809 PMC5792886

[ece373731-bib-0046] Shine, R. 1988. “The Evolution of Large Body Size in Females: A Critique of Darwin's ‘Fecundity Advantage’ Model.” American Naturalist 131, no. 1: 124–131. 10.1086/284778.

[ece373731-bib-0047] Slavenko, A. , N. Cooper , S. Meiri , G. Murali , D. Pincheira‐Donoso , and G. H. Thomas . 2024. “Evolution of Sexual Size Dimorphism in Tetrapods Is Driven by Varying Patterns of Sex‐Specific Selection on Size.” Nature Ecology & Evolution 9, no. 3: 464–473. 10.1038/s41559-024-02600-8. 39715950 PMC11893467

[ece373731-bib-0048] Stephens, D. W. , and J. R. Krebs . 1986. Foraging Theory. Princeton University Press (Monographs in Behavior and Ecology).

[ece373731-bib-0049] Vollrath, F. , and G. A. Parker . 1992. “Sexual Dimorphism and Distorted Sex Ratios in Spiders.” Nature 360, no. 6400: 156–159. 10.1038/360156a0.

[ece373731-bib-0050] Walker, S. E. , and A. L. Rypstra . 2003. “Sexual Dimorphism and the Differential Mortality Model: Is Behaviour Related to Survival?: Sexual Dimorphism, Behaviour and Survival.” Biological Journal of the Linnean Society 78, no. 1: 97–103. 10.1046/j.1095-8312.2003.00134.x.

[ece373731-bib-0051] Wilder, S. M. , and A. L. Rypstra . 2008a. “Sexual Size Dimorphism Mediates the Occurrence of State‐Dependent Sexual Cannibalism in a Wolf Spider.” Animal Behaviour 76, no. 2: 447–454. 10.1016/j.anbehav.2007.12.023.

[ece373731-bib-0052] Wilder, S. M. , and A. L. Rypstra . 2008b. “Sexual Size Dimorphism Predicts the Frequency of Sexual Cannibalism Within and Among Species of Spiders.” American Naturalist 172, no. 3: 431–440. 10.1086/589518. 18616388

[ece373731-bib-0053] Wolff, J. O. , K. Wierucka , G. B. Paterno , et al. 2022. “Stabilized Morphological Evolution of Spiders Despite Mosaic Changes in Foraging Ecology.” Systematic Biology 71, no. 6: 1487–1503. 10.1093/sysbio/syac023. 35289903

